# Tissue-Specific Down-Regulation of the Long Non-Coding RNAs PCAT18 and LINC01133 in Gastric Cancer Development

**DOI:** 10.3390/ijms19123881

**Published:** 2018-12-04

**Authors:** Kobra Foroughi, Mohammad Amini, Amir Atashi, Habibollah Mahmoodzadeh, Ute Hamann, Mehdi Manoochehri

**Affiliations:** 1 School of Medicine, Shahroud University of Medical Sciences, Shahroud 3614773955, Iran; k.forughi@shmu.ac.ir (K.F.); mohammadamini0624@gmail.com (M.A.); 2 Department of Medical Laboratory Sciences, School of Allied Medical Sciences, Shahroud University of Medical Sciences, Shahroud 3614773955, Iran; atashia@shmu.ac.ir; 3 Department of Surgery, Cancer Institute, Tehran University of Medical Sciences, Tehran 6718743161, Iran; hmahmoodzadeh@tums.ac.ir; 4 Molecular Genetics of Breast Cancer (B072), German Cancer Research Center (DKFZ), 69120 Heidelberg, Germany; u.hamann@dkfz-heidelberg.de

**Keywords:** gastric cancer, long noncoding RNA, gene expression, tumor suppressor, GEO and TCGA databases

## Abstract

Gastric cancer (GC) is the fifth most common cancer and the third most frequent cause of cancer deaths worldwide. The high death rate associated with GC, and lack of appropriate biomarkers for diagnosis, prognosis, and treatment emphasize the need for identification of novel molecules. Given the emerging roles for long non-coding RNAs (lncRNAs) in cancer development, we studied novel lncRNA candidates involved in gastric carcinogenesis. LncRNA candidate discovery was performed using analyses of available datasets and literature. Validation was done using an internal sample set of GC/normal tissues, and external independent datasets. Network analysis and functional annotation of co-expressed protein coding genes were performed using the weighted gene correlation network analysis (WGCNA) and ingenuity pathway analysis. Two novel lncRNAs, *PCAT18* and *LINC01133*, associated with GC development were identified by analysis of the discovery Gene Expression Omnibus (GEO) datasets. The down-regulation of these genes in GC tissues was successfully validated internally and externally. The results showed a tissue-specific down-regulation of *PCAT18* and *LINC01133* in gastrointestinal tissues. WGCNA and ingenuity pathway analyses revealed that the genes co-expressed with the two lncRNAs were mostly involved in metabolic pathways and networks of gastrointestinal disease and function. Our findings of a tissue-specific down-regulation of *PCAT18* and *LINC01133* in gastric and other gastrointestinal cancers imply that these lncRNAs may have a tumor suppressive function in the development of these tumor entities. The two lncRNA biomarkers may contribute to a better understanding of the complex mechanisms of gastric carcinogenesis.

## 1. Introduction

Gastric cancer (GC)/stomach cancer is the fifth most common cancer in the world with almost one million new cases reported in 2012 [1]. More than 70% of GCs occur in developing countries and half the world’s total occurs in Eastern Asia. GC is the third leading cause of cancer-related death with the highest estimated mortality rate observed in Eastern Asia. Despite advances in diagnosis, approximately half of all GC patients are diagnosed at an advanced stage and have a poor five -year survival rate of less than 20% [2,3]. Furthermore, surgery and cytotoxic chemotherapy have limited value in advanced disease and markers for targeted therapy are scarce. The etiology of GC is multifactorial involving genetic, epigenetic, and environmental risk factors including *Helicobacter pylori* infection, dietary, lifestyle, and other factors [4,5,6].

Long non-coding RNAs (lncRNAs) participate in various biological processes including cell proliferation, cell differentiation, migration, invasion, and apoptosis, mainly by regulating of gene expression at the epigenetic, transcriptional, post-transcriptional, and translational levels [7,8]. They execute their various biological functions through different mechanisms as transcriptional enhancers, signals, decoys, guides, or as scaffolds for their interacting proteins; DNA or RNA molecules. LncRNAs are also crucial players in the development and progression of cancer [9,10,11]. A dysregulation of these genes was reported in a broad spectrum of tumors [12,13,14], which correlated with the stage and prognosis of several tumor types, and was linked to chemotherapy resistance and targeted therapy [15].

Accumulating evidence has demonstrated that many lncRNAs are also dysregulated in GC and closely related to tumorigenesis, metastasis, prognosis, and diagnosis. Several oncogenic and tumor-suppressive lncRNA genes involved in the development of the disease have been identified [16]. Expression levels of the widely studied oncogenic lncRNAs *H19* and *HOTAIR* have been shown to be associated with different cellular phenotypes and clinical characteristics [17,18]. While expression of the oncoge *BANCR* correlated with tumor invasion depth, metastasis (M) status, lymph node status, and clinical stage in GC patients [19]. Of the tumor-suppressive lncRNAs, *MEG3* correlated with stage, tumor invasion depth, and tumor size [20], whereas expression of *GAS5* correlated with prognosis [21].

The high death rate and the lack of appropriate biomarkers for diagnosis, prognosis, and treatment of GC demand the identification of novel molecules. Given the promising role of lncRNAs in cancer development, the present study aimed to identify novel lncRNAs associated with gastric carcinogenesis. For this purpose, we used lncRNA expression data of GC and normal tissues acquired from public repositories, in addition to a sample set of 25 GC and paired normal tissues. To predict the function of differentially expressed lncRNA candidates, weighted gene correlation network analysis (WGCNA) co-expression network analysis was performed followed by functional pathway enrichment analysis of the co-expressed genes.

## 2. Results

### 2.1. Identification of LncRNA Candidates Associated with Gastric Cancer Using Gene Expression Omnibus (GEO) Datasets and Literature Data

**Figure 1:**
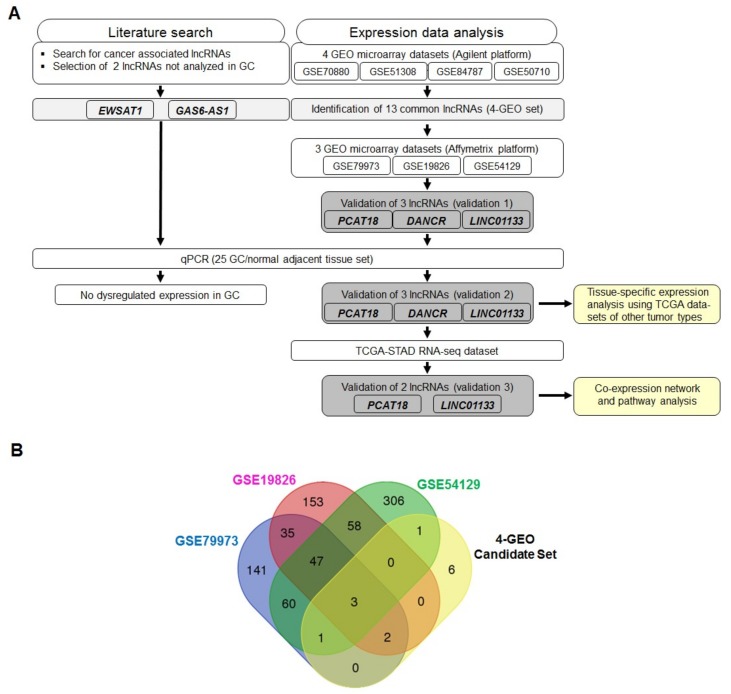
Discovery and selection of long non-coding RNA (lncRNA) candidates. (**A**) Overview of the analysis pipeline; (**B**) Venn diagram showing the number of common and unique lncRNA genes across the GEO datasets. The blue circle represents the lncRNAs found in GSE79973, the pink circle those found in GSE19826, the green circle those found in GSE54129, and the yellow circle the 13 lncRNAs common to the four-GEO set (four Agilent microarray expression datasets of gastric cancer (GC)).

An overview of the analysis pipeline is presented in Figure 1A. Five lncRNA candidates were selected; two, *EWSAT1* and *GAS6-AS1*, were selected based on a comprehensive literature search on novel lncRNAs with no previous association with GC development, but deregulated in other tumor entities (Table S1); and three, *PCAT18*, *DANCR*, and *LINC01133*, were selected based on the analysis of GC datasets from GEO.

**Figure 2:**
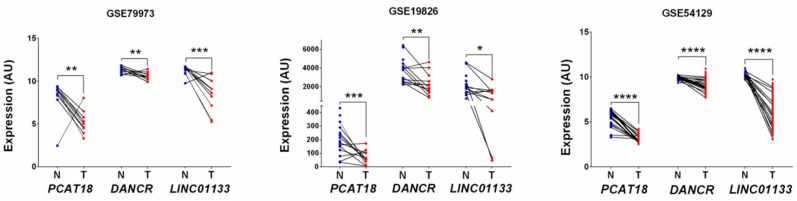
*PCAT18*, *DANCR*, and *LINC01133* expression levels in GC and adjacent normal tissues. Expression data were retrieved from the GEO datasets (**A**) GSE79973, (**B**) GSE19826, and (**C**) GSE54129. N, normal tissue (blue dots); T, tumor tissue (red dots); AU, arbitrary unit. * *p* ≤ 0.05, ** *p* ≤ 0.01, *** *p* ≤ 0.001, **** *p* ≤ 0.0001.

LncRNA expression data analysis of the four GEO discovery datasets resulted in the identification of 13 differentially expressed lncRNAs (four-GEO candidate set; Table 1 and Figure 1A). Of these, 12 lncRNAs were down-regulated (all *p* < 0.05) in GC tissues and one was upregulated (*p* < 0.05) compared to normal tissues. The number of common and unique lncRNAs across the different datasets is shown in the Venn diagram (Figure 1B). Expression of three lncRNAs, *PCAT18, DANCR*, and *LINC01133*, was validated in three other GEO datasets (validation 1; Figure 2 and Table S2).

### 2.2. LncRNA Expression Levels in the 25 GC/Normal Tissue Sample Set and Associations with Clinical, Histopathological, and Epidemiological Parameters

**Figure 3:**
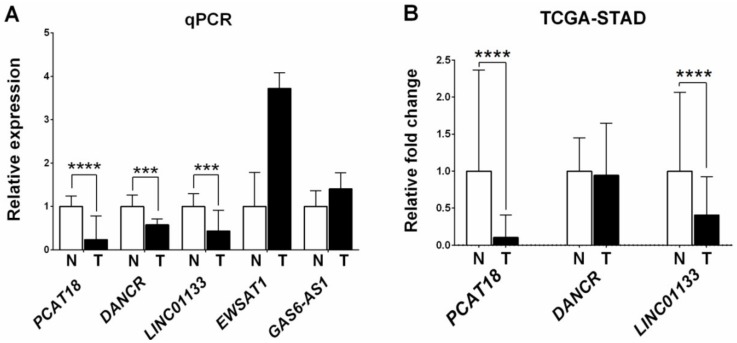
Expression levels of *PCAT18*, *DANCR*, *LINC01133*, *EWSAT1*, and *GAS6-AS1* in GCs and their adjacent normal tissues. (**A**) Expression was measured in 25 GCs and their adjacent normal tissues by real-time qPCR; (**B**) Expression data were retrieved from the TCGA-STAD database. *** *p* ≤ 0.001, **** *p* ≤ 0.0001.

Next we analyzed expression of the five lncRNAs candidates in the 25 GC/normal tissue sample set using real-time quantitative (q) PCR. Expression data were validated for *PCAT18*, *DANCR*, and *LINC01133* (validation 2). All three lncRNAs were down-regulated in GC tissues (*PCAT18: p* ≤ 0.0001; *DANCR: p* ≤ 0.001; and *LINC01133: p* ≤ 0.001) (Figure 3A). No dysregulated expression was observed for *EWSAT1* and *GAS6-AS1* (*p* = 0.1034 and *p* = 0.3049, respectively) and therefore these two lncRNAs were excluded from any further analyses.

Table 2 shows the distribution of the 25 GCs according to selected clinical, histopathological, and epidemiological parameters and associations with *PCAT18*, *DANCR*, and *LINC01133* expression levels. With the exception of gender and smoking status, parameters showed no associations with expression levels. *DANCR* expression was associated with gender (*p* = 0006) and smoking status (*p* = 0.005), whereas *LINC01133* expression was associated with gender (*p* = 0.002). Both *LINC01133* and *DANCR* lncRNA expression levels were higher in females than males. *DANCR* lncRNA expression was higher in non-smokers compared to smokers. *PCAT18* expression was not associated with any of the investigated parameters.

### 2.3. Analysis of LncRNA Expression Levels Using RNA-Sequencing Data from The Cancer Genome Atlas- Stomach Adenocarcinoma (TCGA-STAD)

For further validation TCGA RNA-seq expression data for *PCAT18*, *DANCR*, and *LINC01133* in stomach cancer and normal tissues were analyzed. As shown in Figure 3B, the expression of *PCAT18* and *LINC01133* was down-regulated in stomach cancer tissues (*p* = 6.35 × 10^−19^ and *p* = 0.3.40 × 10^−7^, respectively; validation 3). No deregulated expression was observed for *DANCR*.

The relationship between expression of *PCAT18* and *LINC01133*, overall survival and tumor grade/stage was investigated using the The Cancer Genome Atlas- Stomach Adenocarcinoma (TCGA-STAD) dataset. No statistically significant associations were identified.

**Figure 4:**
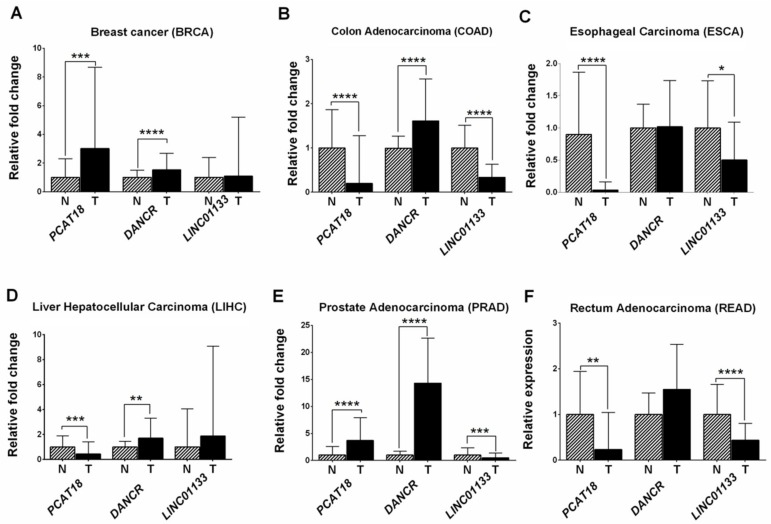
Expression levels of *PCAT18*, *DANCR*, and *LINC01133* in various tumor entities and their adjacent normal tissues. (**A**) Breast cancer (BRCA); (**B**) colon adenocarcinoma (COAD); (**C**) esophageal carcinoma (ESCA); (**D**) liver hepatocellular carcinoma (LIHC); (**E**) prostate adenocarcinoma (PRAD); and (**F**) rectum adenocarcinoma (READ). Expression data were retrieved from TCGA. * *p* ≤ 0.05, ** *p* ≤ 0.01, *** *p* ≤ 0.001, **** *p* ≤ 0.0001.

Further analysis of RNA-seq expression data of these genes in six other cancer entities including four gastrointestinal cancers (esophageal, colon, rectum, and liver), prostate, and breast cancers showed an up-regulation of *PCAT18* in prostate and breast cancers (*p* = 2.96 × 10^−11^ and *p* = 6.33 × 10^−8^, respectively), and a down-regulation in all gastrointestinal cancers (Figure 4). *LINC01133* was also down-regulated in esophageal, colon, and rectal cancers as well as in prostate cancer. In contrast, *DANCR* was up-regulated in breast, prostate, liver, and colon cancers.

### 2.4. Weighted Gene Correlation Network Analysis and Functional Annotation of Co-Expressed Genes

**Figure 5:**
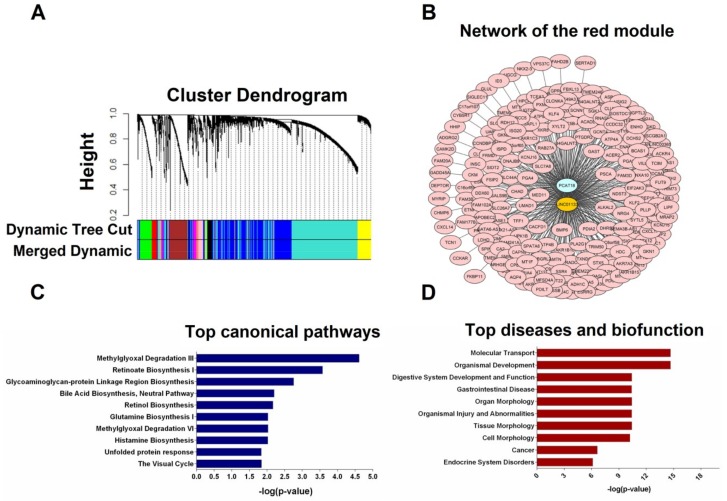
Weighted gene correlation network analysis (WGCNA) and pathway analysis using RNA-sequencing (RNA-seq) data from the TCGA stomach cancer dataset. (**A**) WGCNA cluster dendrogram of differentially expressed mRNAs and lncRNAs. Each leaf (vertical line) in the dendrogram corresponds to a gene. The color row underneath the dendrogram shows the assigned original module and the merged module. The lncRNA candidates *PCAT18* and *LINC01133* were clustered in the red module; (**B**) The lncRNA-mRNA network of the red module visualized using the cytoscape software platform; (**C**) Over-represented terms in the Molecular and Cellular Function category; (**D**) Top diseases and biofunctions related to co-expressed genes.

WGCNA identified 17 co-expression clusters/modules of co-expressed genes in the TCGA-STAD RNA-seq dataset. The two down-regulated lncRNAs *PCAT18* and *LINC01133* were clustered in the red module (Figure 5A). This module contained 218 other genes which are provided in Table S3. The relation between the co-expressed genes of the red module and the lncRNAs is shown in Figure 5B.

To assess how lncRNA genes may contribute to GC development, functional enrichment analysis of the 218 co-expressed genes was performed using Ingenuity Pathway Analysis (IPA). The top ten canonical pathways and the top ten diseases and functions related to the co-expressed genes are shown in Figure 5C,D. The top canonical pathways included different metabolic pathways which play a role in degradation of glycolysis side-product, amino acid biosynthesis, and retinoate biosynthesis. The biological functions of these genes were associated with molecular transport, organismal development, digestive system development and function, organ morphology, and tissue morphology.

## 3. Discussion

Despite advances in diagnosis, prognosis, and treatment, GC remains a worldwide public health concern. Our contribution to this area of investigation was to identify novel lncRNAs involved in gastric carcinogenesis by analyzing gene expression datasets obtained from GC and normal tissues from the GEO and TCGA databases in addition to another sample set of 25 GC and paired normal tissues. While some lncRNAs dysregulated in GC and their clinical value as potential biomarkers for diagnosis and prognosis have been previously reported [22], this study provides additional data on two novel lncRNAs contributing to GC and their potential functions.

LncRNAs are non-coding transcripts longer than 200 nucleotides that do not overlap with annotated coding genes. These transcripts are involved in chromatin remodeling and genome architecture, RNA stabilization and transcriptional regulation [23]. In the present study two lncRNAs down-regulated in GC compared to normal tissues were identified; *PCAT18* and *LINC01133*. The expression data of the lncRNAs are robust, as they were validated in three independent sample sets. This data implies that these lncRNAs may act as tumor suppressors acting during the development of GC.

The prostate cancer associated transcript 18 (*PCAT18*) gene located at 18q11.2 is highly expressed in prostate cancer. Its’ silencing in prostate cancer cells leads to the inhibition of cell proliferation, migration, and invasion [24]. In the present study we observed a down-regulation of *PCAT18* in GC/stomach cancer tissues pointing to a role of this lncRNA in GC development. A down-regulation was also observed in other gastrointestinal cancers including cancers of the esophagus, colon, rectum, and liver, while an up-regulation was observed in breast and prostate cancers. These findings imply that *PCAT18* down-regulation is specific for gastrointestinal tumors. Furthermore, based on normal tissue RNA-seq expression data from the Genotype-Tissue Expression database (GTEx), *PCAT18* is highly expressed in normal stomach tissue [25]. Its high expression suggests a potential regulatory function in this tissue and, when down-regulated, may be a cause or consequence of stomach cancer development.

The long intergenic non-coding RNA 1133 (*LINC01133*) gene on chromosome 1q23.2 is down-regulated in colorectal cancer [26], while an up-regulation was reported in different types of lung cancer [27,28]. In non-small cell lung cancer (NSCLC), its expression inversely correlated with the expression of *KLF*, *P21*, and E-cadherin suggesting an oncogenic function in NSCLC. Furthermore, LINC01133 was shown to sponge the miR-422a to aggravate the tumorigenesis of human osteosarcoma [29]. Our data of a role for *LINC01133* in GC are in line with those from a recent study that showed an association of a reduced *LINC01133* expression with aggressive tumor phenotypes [30]. The authors also showed that this lncRNA inhibits GC progression and metastasis implying its potential use as an anti-metastatic therapeutic target for this disease. Similar to *PCAT18*, *LINC01133* is also highly expressed in normal stomach tissues based on GTEx data implying that its deregulation in normal stomach tissue may play a role in the fate of cells and cancer progression.

Differentiation antagonizing non-protein coding RNA (*DANCR*) located at 4q12 is up-regulated in various cancers including hepatocellular carcinoma [31,32], colorectal cancer [33], prostate cancer [34], osteosarcoma [35], and stomach cancer [36]. In contrast to the previous stomach cancer study, a down-regulation of *DANCR* in GC was found in the present study using the GEO and qPCR data, which however was not validated in the TCGA dataset. Thus, further studies on the expression of this lncRNA in GC and its function are warranted.

The gene co-expression network analysis using WGCNA was performed to identify modules containing *PCAT18*, *LINC01133*, and their co-expressed genes. Seventeen modules were identified, one of which was containing both lncRNAs and 218 eigengenes. Pathway analysis revealed that the top canonical pathways were mostly related to various cell metabolic pathways. Given that tumor cells often have an altered metabolism to cope with the demand of cell-mass increase during growth [37], these lncRNAs may be involved in the control of some metabolic pathways in GC cells. Among the networks, the top ones were linked to molecular transport, organismal development, and gastrointestinal disease. The functional annotations of these top networks were associated with various biofunctions and diseases of the stomach. Merging of the top four networks identified the extracellular-signal-regulated kinase/mitogen-activated protein kinases (ERK/MAPK) pathway as a hub with more connections to other co-expressed genes. It was reported that abnormal activation and mutations of genes involved in the ERK/MAPK pathway occur in more than 50% of human cancer types [38]. Recently, various studies have shown that the ERK/MAPK pathway is involved in regulating cellular mobility in GC cell lines suggesting that this pathway influences GC cell migration and invasion [39]. Besides, of the top upstream regulators, the homeobox genes *CDX1* and *CDX2* have been reported to be crucial players in stomach carcinogenesis [40], while *XBP1* was shown to control the maturation of gastric zymogenic cells [41].

To elucidate the mechanisms of how these lncRNAs exert their functions, lncRNA–lncRNA interactions were predicted in silico using lncRNA2-target databases. An interaction between PCAT18 and BANCR was predicted. *PCAT18* expression was increased following knock-down of *BANCR* [42]. Interestingly, a recent study on GC reported that *BANCR* was significantly up-regulated in GC tissues, and cell lines and its down-regulation led to the inhibition of GC cell proliferation [43]. Accordingly, down-regulation of *PCAT18* along with up-regulation of *BANCR* in GC tissues suggests a possible regulatory interaction between these two lncRNAs. Further exploration using AnnoLnc [44], a web server, which provides systematic annotation of newly identified human lncRNAs, predicted an interaction of PCAT18 with C15orf57 (now called *CCDC32* gene) and GABRR3 protein.

Our study has some limitations that are related to the small size of the internal validation set, and to the retrospective study cohorts. Another limitation is the lack of functional analyses, which should be performed to yield detailed insight into the mechanism of downregulation of *PCAT18* and *LINC01133* in gastric carcinogenesis.

Altogether, we showed a decreased tissue-specific expression of *PCAT18* and *LINC01133* lncRNAs in GC and other gastrointestinal tumor tissues, suggesting a role of these lncRNAs in the development of gastrointestinal tumors. The reduced lncRNAs expression levels may interfere with normal harmony of gene regulation in normal gastric cells and potentiate them towards GC progression and development, which may be achieved via a gene regulation process leading to metabolic adaptation in tumor cells. The two lncRNA biomarkers may contribute to a better understanding of the complex mechanisms of gastric carcinogenesis. The reported data should guide future studies on the associations of *PCAT18* and *LINC01133* with GC and their functions.

## 4. Materials and Methods

### 4.1. Data Extraction from the GEO Database and Literature Review

Ten GC datasets were retrieved from GEO (http://www.ncbi.nlm.nih.gov/geo/) using the keywords: “lncRNA stomach cancer” (study keyword), “homo sapiens” (organism), “expression profiling by array” (study type), and “tissue” (attribute name). Seven datasets fulfilling the following parameters were selected for expression analyses: (1) Availability of data on GC and adjacent normal tissues; (2) inclusion of expression data of lncRNA genes; and (3) availability of minimum information about the microarray experiment. Four datasets (obtained using the Agilent platform);, GSE70880 (20 tumor and 20 adjacent normal tissues), GSE51308 (5 tumor and 5 adjacent normal tissues), GSE84787 (10 tumor and 10 adjacent normal tissues), and GSE50710 (10 tumor and 10 adjacent normal tissues) were selected for the discovery of lncRNA candidates. These sets contained data from 45 GCs and their paired normal tissues. Besides, three datasets (obtained using the Affymetrix platform); GSE79973 (10 tumor and 10 adjacent normal tissues), GSE19826 (12 tumor and adjacent normal tissues + 3 normal gastric tissues), and GSE54129 (111 tumor and 21 noncancerous gastric tissues), were used for data validation. These sets contained data from 133 GCs and 46 noncancerous normal tissues.

The comparison between tumor and adjacent normal tissues allowed the identification of differentially expressed genes in the GEO datasets. *p* values were adjusted (*p*_adj_.) using the Benjamini and Hochberg method. A *p*_adj_. < 0.05 and a |logFC| ≥ 1 were set as cut-off criteria [45,46,47]. Among the top candidates, those already known to be associated with GC were excluded.

Moreover, in order to identify novel lncRNAs associated with other cancers but not reported in GC, a comprehensive PubMed literature search was performed. The following keywords, selected from the medical subject headings (MeSH) database, were used: (“Neoplasms”) AND “RNA, Long Noncoding”).

### 4.2. Patient Samples

Fifty tissues comprising 25 GC and paired normal tissues (25 GC/normal tissue sample set) were obtained from the Iran National Tumor Bank (INTB, Tehran, Iran). All tissues were collected during surgical resection of patients diagnosed with primary GC at the Imam Khomeini Hospital, Tehran, Iran from 02/2009 to 11/2014. Adjacent normal tissues were obtained from areas at least 6 cm away from the tumor site. None of the patients received radiation and/or chemotherapy treatment before surgery. Tissues were stored in liquid nitrogen until nucleic acid extraction.

Data on selected clinical, histopathological, and epidemiological parameters including age (<60, ≥60 years), gender, site of primary tumor (gastric cardia, antrum, stomach), tumor size (<5, ≥5 cm), histological grade (1–4), lymph node status (N0, ≥N1), vascular invasion (yes, no), perineural invasion (yes, no) serosal invasion (yes, no), clinical stage (1–4), family history of GC (yes, no), and smoking status (ever, never) are presented in Table 2.

The study was approved by the Ethical Committee of the Shahroud University of Medical Sciences (9559, 08/10/2016). All study participants provided written informed consent.

### 4.3. RNA Extraction and cDNA Synthesis

Tissues were grinded in liquid nitrogen using a mortar and pestle, instantly transferred into the lysis buffer, and homogenized using a needle and syringe. Total RNA was extracted using AllPrep DNA/RNA Mini kit (Qiagen, Hilden, Germany), according to manufacturer’s instructions. The quantity and quality of isolated RNA samples were determined by Picodrop microliter spectrophotometer (OEM, Hinxton, UK), and electrophoresis on a 0.8% agarose gel. Afterwards, 1 μg of total RNA was converted into cDNA using PrimeScript^TM^ RT reagent kit (TaKaRa Bio, Shiga, Japan) according to the manufacturer’s instruction.

### 4.4. Real-Time PCR

Real-Time qPCR was performed using the Bio-Rad CFX96^TM^ Real-Time System (Bio-Rad, Foster City, CA, USA). The 20 μL reaction contained 10 μL SYBER Master Mix without ROX (Ampliqon, 5230 Odense M, Denmark), 8 μL ddH_2_O, 1 μL cDNA template, 5 μM of each primer (forward and reverse). The real time qPCR primer sequences are provided in the Table S4. Conditions for amplification were 95 °C for 15 min, followed by 40 cycles of 95 °C for 5 s, 60 °C for 30 s. Melting curves were obtained by slow heating (0.5 °C/s) at temperatures in the range of 65 to 95 °C. All samples were run in duplicate.

### 4.5. Data Extraction from TCGA and Data Analyses

For further data validation, RNA-sequencing data (RNA-seq) of stomach (gastric) adenocarcinoma (STAD) from TCGA were analyzed and used for comparison. Moreover, the expression levels of the lncRNA candidates were analyzed in other cancer entities including adenocarcinomas of the colon, esophagus, rectum, liver hepatocellular, prostate, and breast and adjacent normal tissues. The RNA-seq raw data files were downloaded from the TCGA GDC data portal, normalized, and filtered using the R/Bioconductor software package TCGAbiolinks (R version 3.4.2, http://www.r-project.org/) [48]. Differential expression analysis of lncRNA genes in tumor and normal tissues was performed using the edgeR package [49]. This package implements the trimmed mean of M-values (TMM) method to give the normalized read counts.

### 4.6. Weighted Gene Correlation Network Analysis

Given that the functions of most lncRNAs are unknown, prediction of their functions mostly relies on the analysis of their co-expressed genes. Network analysis was performed using the WGCNA package in R as described previously [50,51]. To identify modules of highly correlated genes, WGCNA was performed on RNA-seq data of the TCGA-STAD dataset obtained from 407 GC and normal tissues. To identify modules with different expression patterns, a soft threshold power was assigned to create co-expression networks. The networks were built by merging genes with highly similar co-expression patterns into modules and the eigengenes of these modules were determined. Finally, the module with the key lncRNAs and their co-expressed genes was obtained. The reconstructed co-expression network was visualized using the Cytoscape software (version 3.5.1 (http://www.cytoscape.org)) [52].

### 4.7. Functional Annotation of the Co-Expressed Genes in the Module

To investigate the potential functions of *PCAT18* and *LINC01133* and their associated biological pathways, a functional enrichment analysis of their co-expressed genes was performed using Ingenuity Pathway Analysis (IPA; Ingenuity Systems, Mountain View, CA, USA) software. IPA provides a graphical representation of the molecular relationships between genes in networks. Functional analysis identified statistical significant (Fisher’s exact test *p* value < 0.05) over-represented Canonical Pathways, Molecular and Cellular Functions, Physiological System Development and Function, upstream regulators, and Diseases and Bio Functions in the imported data sets.

### 4.8. Statistical Analyses

Statistical analyses were performed using GraphPad Prism 6 software (GraphPad Software Inc., San Diego, CA, USA) and SPSS 24.0 (SPSS Inc., Chicago, IL, USA). Differences between the means of two groups were determined using student’s *t*-test. All *p* values were two-sided, with a *p* value of less than 0.05 considered statistically significant. All results are presented as the mean ± standard deviation (SD) of the experiments.

## 5. Conclusions

The down-regulation of *PCAT18* and *LINC01133* in GC implies that these lncRNAs may have a tumor suppressive function in the development of gastric tumors. The two lncRNA biomarkers may contribute to a better understanding of the complex mechanisms of gastric carcinogenesis.

## Figures and Tables

**Table 1:** Differentially expressed lncRNAs in gastric cancer based on analyses of the four discovery GEO datasets.

Gene ID *	Gene Symbol	Gene Name	Log2FC	*p* Value	Regulatory Status
728606	*PCAT18*	Prostate cancer associated transcript 18	−2.62	1.58 × 10^−4^	Down
57291	*DANCR*	Differentiation antagonizing non-protein coding RNA	−0.86	1.37 × 10^−3^	Down
283738	*NTRK3-AS1*	Neurotrophic receptor tyrosine kinase 3 antisense RNA 1	1.24	2.0 × 10^−4^	Up
101928223	*LINC01612*	Long intergenic non-protein coding RNA 1612	−1.50	2.4 × 10^−4^	Down
404201	*WDFY3-AS2*	WD repeat and FYVE domain containing 3 antisense RNA 2	−0.77	8.85 × 10^−4^	Down
100505875	*LINC01088*	Long intergenic non-protein coding RNA 1088	−2.69	4.44 × 10^−9^	Down
102723487	*LINC01497*	Long intergenic non-protein coding RNA 1497	−1.42	1.82 × 10^−7^	Down
100505633	*LINC01133*	Long intergenic non-protein coding RNA 1133	−1.42	9.32 × 10^−3^	Down
348761	*SPATA3-AS1*	Spermatogenesis associated 3 antisense RNA 1	−1.96	2.02 × 10^−9^	Down
401172	*LINC02145*	Long intergenic non-protein coding RNA 2145	−1.95	3.79 × 10^−9^	Down
100507099	*FRY-AS1*	FRY microtubule binding protein antisense RNA 1	−1.30	0.02946	Down
286190	*LACTB2-AS1*	Lactamase beta 2 antisense RNA 1	−1.30	0.02946	Down
101927171	*LINC01564*	Long intergenic non-protein coding RNA 1564	−3.30	0.03868	Down

Log2FC < 0: down-regulated, Log2FC > 0: up-regulated. * From NCBI RefSeqGene.

**Table 2:** Associations of clinical and histopathological tumor parameters with *PCAT18*, *DANCR*, and *LINC01133* expression levels.

Parameter	Cases *n* (%)	*p* Value		
		*PCAT18*	*DANCR*	*LINC01133*
**Age (years)**				
<60	12 (48)	0.623	0.965	0.504
≥60	13 (52)			
**Gender**				
Male	21 (84)	0.856	**0.006**	**0.002**
Female	4 (16)			
**Site of primary tumor**				
Gastric cardia	4 (16)	0.758	0.359	0.536
Antrum	5 (20)			
Stomach	16 (64)			
**Tumor size (cm)**				
<5	11 (44)	0.597	0.809	0.666
≥5	12 (48)			
Unknown	2 (8)			
**Histological grade**				
1, 2	17 (68)	0.316	0.879	0.982
3, 4	8 (32)			
**Lymph node status**				
N0	19 (76)	0.404	0.831	0.771
≥N1	6 (24)			
**Vascular invasion**				
Yes	19 (76)	0.404	0.831	0.771
No	6 (24)			
**Perineural invasion**				
Yes	16 (64)	0.966	0.843	0.938
No	9 (36)			
**Serosal invasion**				
Absent	11 (44)	0.243	0.913	0.732
Present	14 (56)			
**Clinical stage**				
1, 2	9 (36)	0.223	0.374	0.221
3, 4	16 (64)			
**Family history of gastric cancer**				
Yes	9 (36)	0.798	0.663	0.431
No	16 (64)			
**Smoking status**				
Ever	14 (56)	0.056	**0.005**	0.057
Never	11 (44)			

Significant *p* values are marked in bold.

## Supplementary Materials

Supplementary materials can be found at http://www.mdpi.com/1422-0067/19/12/3881/s1.

## Abbreviations

GC	Gastric Cancer
GEO	Gene Expression Omnibus
TCGA	The Cancer Genome Atlas
lncRNAs	Long Non-Coding RNAs
WGCNA	Weighted Gene Correlation Network Analysis
RNA-seq	RNA-Sequencing
STAD	Stomach Adenocarcinoma
TMM	Trimmed Mean of M-Values
IPA	Ingenuity Pathway Analysis
SD	Standard Deviation
PCAT18	Prostate Cancer Associated Transcript 18
LINC01133	Long Intergenic Non-Coding RNA 1133
DANCR	Differentiation Antagonizing Non-Protein Coding RNA
GTEx	Genotype-Tissue Expression Database
NSCLC	Non-Small Cell Lung Cancer
